# Alcoholic brain injury is a modifiable risk factor: natural active substance intervention promises to improve alcohol-induced cognitive impairment

**DOI:** 10.3389/fnut.2025.1578169

**Published:** 2025-10-01

**Authors:** Wenqi Wang, Yuhan Du, Xuewen Tong, Jiahua Liu, Mingming Lei, Liankui Wen, Yang He, Xinxin Meng, Xuanwei Xu

**Affiliations:** ^1^Department of Food Science and Engineering, Jilin Agricultural University, Changchun, China; ^2^Jilin Forest Industry Group Quanyangquan Beverage Co., Ltd., Changchun, China; ^3^Instrument and Equipment Management Services Testing Center, Jilin Agricultural University, Changchun, China

**Keywords:** alcohol, cognitive impairment, natural substances, gut-brain axis, mechanism

## Abstract

Alcohol-related brain injury is often manifested as cognitive decline, accompanied by gut microbiota dysbiosis and disruptions in neuroimmune regulation. According to the gut-brain axis theory, natural compounds may alleviate alcohol-induced brain damage by modulating the gut microbiota. This review summarizes the differential vulnerability of various brain regions to alcohol-induced damage and highlights recent advances in the regulatory effects of natural compounds—including polysaccharides, polyphenols, and saponins—on the gut microbiota and its key metabolites, such as short-chain fatty acids (SCFAs), bile acids, and neurotransmitters. Particular attention is given to how these microbiota-mediated changes influence central nervous system function through the “gut-nervous-brain axis,” “gut-immune-brain axis” and “gut-endocrine-brain axis,” especially in regions such as the prefrontal cortex (PFC), central amygdala (CeA), paraventricular nucleus of the hypothalamus (PVH), and parietal cortex (PC). Studies indicate that structurally specific polysaccharides, such as those containing β-(1 → 3)-glucan branches, exert neuroprotective effects by promoting the production of key neuroactive metabolites. This review provides a theoretical basis for the application of gut microbiota-targeting natural products in the prevention and treatment of alcohol-related cognitive disorders and highlights their translational potential in brain health interventions.

## Graphical abstract

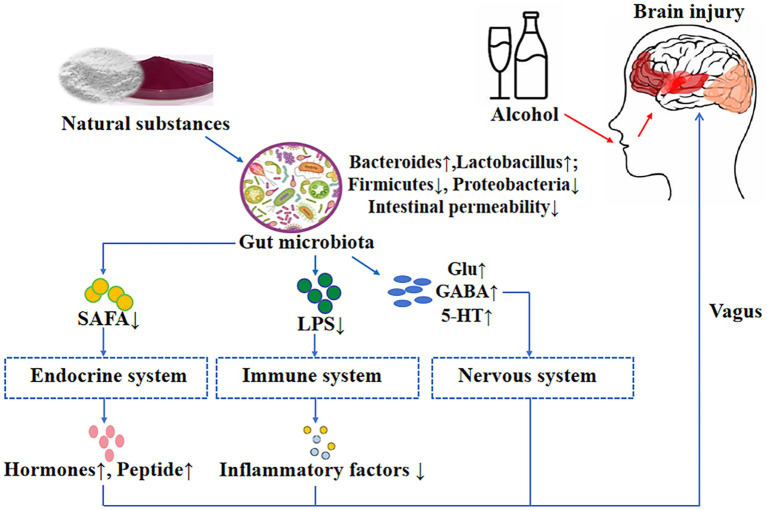

## 1 Introduction

Alcohol, as one of the most widely consumed psychoactive substances globally, has garnered sustained attention in public health research due to its multifaceted impacts. Notably, the consistent upward trend in global alcohol consumption from 2003 to 2018 with projections indicating continued growth (1). Alcohol is a central nervous system depressant, exerts wide-ranging effects on emotion, behavior, attention, and memory. Moreover numerous alcohol-related disorders are associated with brain injury (2). In the central nervous system, hippocampus, amygdala, hypothalamus, frontal lobe and cerebellum have different functions due to distinct regions and structures (3). Structural brain damage due to chronic alcohol use has been widely reported. Brain imaging reveals alcohol’s effects: gray (GM) and white matter (WM) volumes of frontal lobe, hippocampus, thalamus, amygdala and cerebellum were decreased in multiple regions of alcohol use disorder (AUD) patients. Among the brain structures most affected by alcohol are the prefrontal cortex and hippocampus (4). Currently, research has three limitations: (1) The correlation between alcohol exposure and distinct brain regions, as well as that between alcohol-induced brain damage and specific flora, remains elusive; (2) The key mechanisms governing the association between the specific structure of natural active substances and flora lack systematic elucidation; (3) Research on the synergistic regulatory effects of natural active substances on alcohol-related flora disorders and multi-brain-region damage, along with their target associations, is inadequate.

Previous studies on alcohol-induced brain damage have focused on a single signaling pathway such as TLR4/MAPK/NF-κB (5, 6). With the deepening of research on gut-brain axis, it is comprehensively found that the imbalance of gut microbiota may be the starting point of alcohol-induced cognitive impairment (7). This aligns with broader findings in neuropsychiatric disorders, where the gut-brain axis mediates metabolic and inflammatory crosstalk (8). It was found that alcohol caused brain damage in neural, immune and endocrine pathways (9). Excessive alcohol intake increases intestinal permeability and reduces the abundance of beneficial bacteria in the intestine, which reduces neurotransmitter production and increases the level of lipopolysaccharide (LPS), a pro-inflammatory metabolite of bacteria, activates microglia, up-regulates TLR4/MyD88/NF-κB/p65 pathway, and up-regulates tumor necrosis factor-α (TNF-α) and other pro-inflammatory factors (10). Meanwhile, the reduction of beneficial bacteria reduces the production of SCFA, reduces the secretion of intestinal hormones, and inhibits interferon-γ signaling (11). Additionally, alcohol-induced cognitive dysfunction may involve neurochemical imbalances analogous to those observed in other neurodevelopmental and psychiatric conditions: a meta-analysis of neurometabolite levels in individuals with autism spectrum disorder highlighted significant disruptions in brain metabolites linked to cognitive impairment (12), suggesting that similar perturbations—such as altered neurotransmitter precursors or oxidative stress markers—could contribute to alcohol-related deficits. Despite these insights, a major unresolved issue remains: research on the integrated neural, immune, and endocrine mechanisms underlying alcohol-induced cognitive dysfunction, grounded in the gut-brain axis theory, is still incomplete. For example, how neural pathway disruptions (like neurotransmitter imbalances) interact with immune activation (such as microglial overactivity) and endocrine changes (like HPA axis dysregulation) to collectively drive cognitive decline is not fully understood. This represents a critical research frontier that demands attention, as addressing it is essential to developing a holistic understanding of alcohol’s neurobiological effects.

In the past, drugs such as disulam and naltrexone were the main treatments for alcoholism, but most drugs had relatively large side effects (13). Natural bioactive compounds exert their effects through core mechanisms such as antioxidant, anti-inflammatory, and anti-apoptotic actions, involving signaling pathways including TLR4/MyD88/NF-κB, NLRP3, Nrf2/HO-1, BDNF-TrkB, etc. These compounds have low toxicity and high biocompatibility, making them highly promising for the prevention and treatment of alcohol-induced brain injury. Notoginsenoside R1 was reported by Zhang et al. (14–16) to inhibit activation of the TLR4/MyD88/NF-κB signaling pathway, reduce levels of proinflammatory cytokines such as IL-1β, TNF-α, and IL-6, and effectively alleviate neuroinflammatory responses after cerebral ischemia–reperfusion. In addition, plant compounds such as curcumin and berberine can downregulate TLR4/NF-κB signaling while upregulating brain-derived neurotrophic factor (BDNF) expression, thereby inhibiting activation of the NLRP3 inflammasome (17). Research by Scuto et al. (18, 19) indicates that low-dose polyphenols—particularly when combined with probiotics—can synergistically activate key cellular protective pathways such as Nrf2/HO-1 and Sirt1. This effectively alleviates inflammation and oxidative stress, thereby delaying the progression of neurodegenerative diseases. Research has found that gentianin R1 and ginsenoside CK can effectively reduce apoptosis and improve inflammation by inhibiting caspase activity or regulating the expression of glucose metabolism-related proteins (14–16, 20). Previous studies focused mainly on intracellular signaling pathways in neurons, but recent evidence suggests that alcohol first disrupts the intestinal barrier, causing dysbiosis and lipopolysaccharide (LPS) translocation into the bloodstream, which activates the nervous system, immune system, and endocrine system, ultimately leading to brain damage. Research into the mechanisms of alcohol-related brain injury is undergoing a paradigm shift from a “brain-centric” view to an “intestinal origin.”

An intriguing finding is that substances such as polysaccharides and polyphenols, due to their specific structures, can influence the composition of the gut microbiota, reduce intestinal permeability and inflammatory responses, thereby regulating systems including the nervous and immune systems and improving brain dysfunction (21, 22). Due to the 1 → 3 bond connections in polysaccharide side chains, they can increase the abundance of Bacteroides species in the gut, promote short-chain fatty acid production, and regulate the endocrine system (23). Polyphenols with small molecular weight and hydroxyl structure increase the abundance of *Prevotella*, reduce the level of inflammatory factor IL-1β, and improve the effect of neuroinflammation on central immune pathways (24). To identify effective natural substances for alcohol-induced brain damage, future research should explore the relationships between the structural features of natural compounds, gut microbiota modulation, and downstream neural/immune mechanisms. Such investigations could leverage advances in neuroimaging and neurophysiological assessment (25) to quantify brain function changes. Additionally, understanding neural plasticity and resilience—how adaptive responses to stressors enhance brain repair capacity (26)—may inform strategies to promote recovery from alcohol-induced neurodamage. Against this backdrop, the present study is designed to identify natural substances with the potential to ameliorate alcohol-induced brain damage through a systematic analysis of two pivotal relationships: the intricate interplay between the structural features of natural compounds and their specific modulatory effects on gut microbiota composition and function, and the mechanistic connections linking gut microbiota dynamics to perturbations in neural, immune, and endocrine pathways. By unraveling these complex associations, this research endeavors to construct a robust theoretical framework and provide empirical references for the development of targeted protective strategies against alcohol-induced brain injury, ultimately delivering actionable solutions to alleviate the neurocognitive impairments arising from alcohol exposure.

## 2 Alcohol-induced injury to functional parts of the brain

Alcohol (CH_3_CH_2_OH) is a small molecule alcohol compound containing -OH (hydroxyl group) attached to a carbon atom. After ingestion, alcohol is absorbed into the bloodstream by the intestines and stomach. Most of the absorbed alcohol is cleared via the oxidative metabolic pathway through the bloodstream, and a small portion of the absorbed alcohol is metabolized via the non-oxidative metabolic pathway. Alcohol metabolism depends on different enzymes: alcohol dehydrogenase (ADH), aldehyde dehydrogenase (ALDH), catalase and the microsomal ethanol oxidation system (MEOS) containing cytochrome P4502E1 (27). Alcohol and its metabolites can damage the body in many ways through direct or indirect ways, and the damage to the central nervous system is more serious. Alcoholism can lead to irreversible changes in brain nerve tissue. The pathway of alcohol metabolism in the brain is slightly different from that of the liver, and the main pathway involved in alcohol metabolism is through the use of catalase enzymes present in peroxisomes and cytochrome P4502E1. Cytochrome P4502E1 is a variant of cytochrome P450 capable of efficiently oxidizing ethanol in the brain. Studies have found that cytochrome P4502E1 exists and has specific activity in cerebellum, cerebral cortex, thalamus and hippocampus (28). In the brain, cytochrome P4502E1 and catalase convert alcohol to acetaldehyde and hydrogen peroxide, which is converted to acetaldehyde by catalase. This is a highly reactive process that produces highly toxic by-products. Ethanol and acetaldehyde metabolism can lead to damage to functional areas of the brain, resulting in abnormalities in areas involved in cognition, learning, emotion, and executive function. Based on this, this study takes the key mediating role of gut microbiota as the core, analyses the relationship between different alcohol-damaged parts in the brain and their corresponding functions, and further provides a targeted area for the treatment of future brain dysfunctions.

### 2.1 Frontal lobe

The frontal lobe is the part of the cerebral hemisphere before the central sulcus and above the lateral sulcus of the brain, which can be divided into the dorsolateral, medial and basal surfaces. It is an important part of the body that dominates the body’s movement, memory, and autonomous behavior. The prefrontal cortex (PFC), which is located in the anterior region of the frontal lobe and before the motor cortex, is the most complex and highly developed neocortical region (29). The PFC integrates information from other cortices and, from a neuropsychological perspective, acts as executive regulation in the brain. It also mediates higher-order processes required for voluntary goal-directed behavior, among them decision making, inhibitory control, memory, and flexible cognition (30). Structural magnetic resonance studies have shown that the frontal lobe volume of alcoholics is reduced. Measurement of regional cerebral blood flow (CBF) has found a significant decrease in frontal lobe blood flow and a reduction in the amplitude of related potentials. Positron emission tomography (PET) has indicated a significant decline in the frontal lobe glucose metabolic rate (4). Notably, progress has been made in neuroimaging and neurophysiological assessment methods. For example, near-infrared spectroscopy can be used to evaluate brain function (25). These suggest that alcohol may damage the frontal lobe of the brain. Notably, the functional integrity of the prefrontal cortex (PFC) is closely associated with its connectivity to other brain regions. This mechanism might further account for the cognitive and behavioral deficits caused by alcohol. A multi-modal study by Liang et al. (31) explored the role of the prefrontal cortex-basolateral amygdala pathway in cognitive impairment in schizophrenia, emphasizing that the disruption of this neural circuit is crucial for mediating deficits in executive function and memory. This finding is highly relevant to alcohol-induced PFC damage: chronic alcohol exposure may not only directly damage the PFC itself but also disrupt its functional connection with the amygdala (e.g., through neurotransmitter imbalance or inflammatory damage), thus exacerbating dysfunctions in decision-making, emotional regulation, and impulse control—the core symptoms of alcohol use disorders.

Alcohol impairment is associated with structural, physiological and behavioral deficits related to the prefrontal cortex (30). Central stress hormone and neuropeptide signaling pathways have become a bridge between chronic alcohol exposure and pattern tendencies such as affective and cognitive disruption. Concurrently, oxidative stress and neurotransmitter dysregulation disrupt synaptic transmission, causing cellular damage in the PFC. This triggers neuronal dendritic degeneration, impairing synaptic plasticity and memory, and ultimately results in deficits in short-term memory, problem-solving, and impulse inhibition—driving further brain degeneration (32). These pathological processes in the prefrontal cortex are closely related to the gut microbiota, which is a key component of the nervous system-related microbial community. Alcohol-induced gut microbiota dysbiosis (manifested as an imbalance in the abundance of specific bacterial taxa) can affect the release and function of stress-related hormones and neuropeptides in the brain through regulating central stress hormone and neuropeptide signaling pathways, with metabolites such as short-chain fatty acids and tryptophan derivatives, exacerbating the affective and cognitive impairments caused by chronic alcohol exposure. At the same time, gut microbiota dysbiosis also promotes systemic inflammation and endotoxemia, increases oxidative stress in the prefrontal cortex, disrupts neurotransmitter homeostasis and synaptic transmission, leading to cell damage and neuronal dendritic degeneration, highlighting the crucial role of the microbiota in mediating alcohol-induced prefrontal cortex damage (33).

Alcohol exposure activates immune responses, partially activating frontal cortical microglia, increasing levels of pro-inflammatory factors (e.g., TNF-α, IL-1β), and upregulating the NF-κB signaling pathway to induce inflammation. Notably, recent research confirms that the gut microbiota, via the bidirectional gut-brain axis regulatory network, has emerged as a critical mediator of alcohol-induced exacerbation of PFC damage. Alcohol-induced gut microbiota dysbiosis—characterized by reduced microbial diversity and imbalanced community structure—affects PFC function through two core pathways: (1) Dysbiosis disrupts intestinal barrier integrity, for instance, alcohol consumption leads to a significant increase in the relative abundance of harmful bacteria (e.g., Proteobacteria, Enterobacter, Streptococcus) while decreasing levels of beneficial bacteria (e.g., Bacteroidetes, Akkermansia, Faecalibacterium), an imbalance that enables massive translocation of lipopolysaccharide (LPS) (a component of Gram-negative bacterial cell walls) into the bloodstream; circulating LPS reaches the central nervous system, activates the TLR4 signaling pathway in PFC microglia, and amplifies NF-κB-mediated neuroinflammatory responses—synergizing with alcohol’s direct pro-inflammatory effects to exacerbate pro-inflammatory factor accumulation and neuronal damage within the PFC, with research showing that in alcohol users, the abundance of *Faecalibacterium prausnitzii* (a key anti-inflammatory bacterium) is severely reduced, promoting inflammatory environments in both the gut and PFC. (2) Additionally, alcohol-induced dysbiosis significantly reduces the abundance of short-chain fatty acid (SCFA)-producing bacteria (e.g., *Faecalibacterium prausnitzii*, *Roseburia*, *Bifidobacterium*) (111), with long-term alcohol consumption causing substantial declines in gut Bifidobacterium (up to 70% in individuals drinking for ≥3 years); as key gut microbial metabolites, SCFAs normally protect PFC structure and function by inhibiting excessive microglial activation and enhancing blood–brain barrier (BBB) stability, and their depletion weakens this protective mechanism, exacerbating structural damage (e.g., reduced PFC gray matter volume) and executive function deficits (e.g., impaired decision-making) by disrupting synaptic plasticity and neurotransmitter balance (111). Moreover, alcohol disrupts gut microbiota balance in other ways, such as reducing overall microbial diversity—long-term alcohol-exposed individuals exhibit markedly fewer bacterial species than non-drinkers—further contributing to gut-brain axis dysregulation and subsequent PFC impairment.

Excessive or intermittent alcohol exposure disrupts the hypothalamic-pituitary-adrenal (HPA) axis, impairing central-systemic stress feedback regulation. Alcohol enhances forebrain glucocorticoid signaling, directly damaging PFC structure and function (34). Importantly, endocrine hormone-mediated and immune-inflammatory mechanisms of alcohol-induced PFC damage—both influenced by gut microbiota dynamics—may cross-link, collectively promoting frontal lobe impairment. Alcohol-related changes in gut microbiota, such as the altered abundance of certain bacteria, can in turn affect the endocrine system (35). The gut microbiota plays a role in modulating the body’s stress response, and alcohol-induced dysbiosis may disrupt this delicate balance, further aggravating the impact on the HPA axis and ultimately contributing to frontal lobe damage.

### 2.2 Hippocampus

The hippocampus is a structure composed of pyramidal cells located in the subcortex of the cerebral cortex in the limbic system and contains two regions: the corpus ammoniosum angle (CA) and the dentate gyrus (DG). Among them, CA is further divided into CA1, CA2, CA3 and CA4 (36), which are mainly responsible for learning, memory consolidation and memory transmission (37). In rodents, chronic ethanol depletion leads to a reduction in the number of CA1 and CA3 pyramidal neurons, mossy fiber-CA3 synaptic neurons, DG granule cells and local circuit interneurons, with the extent of neuron loss ranging from 10 to 40% (38). Alcohol toxicity causes structural damage to the hippocampal tissue and dysregulation of its associated signaling pathways, in particular leading to massive loss of hippocampal neurons and functional abnormalities (38). Alcohol is responsible for abnormal hippocampal neuronal function in newborn neurones by altering the structural plasticity of hippocampal neurones, dentate granule cells or pyramidal neurones (39). Notably, beyond the well-established neuroinflammatory mechanisms (40, 41), emerging evidence suggests that alcohol-induced hippocampal damage may also involve disruptions in cellular homeostatic processes such as autophagy and mitochondrial function. A recent study revealed that METTL3-dependent m^6^A modification of SNAP29 can induce an “autophagy-mitochondrial crisis” in ischemic microenvironments, characterized by dysregulated autophagic flux and mitochondrial dysfunction. Given that alcohol exposure triggers cellular stress responses in hippocampal neurons (including CA1 and CA3 pyramidal cells), it is speculated that similar mechanisms-namely, alcohol disrupts the m^6^A modification of key regulatory proteins (such as SNAP29) to disrupt the cross-regulation between autophagy and mitochondria-may also be one of the causes of neuronal damage in these regions. This crisis can further exacerbate neuroinflammation by releasing mitochondrial damage-associated molecular patterns (DAMPs), forming a vicious cycle that amplifies the loss of neurons in the CA1 and CA3 regions (42). Based on the above literature, neuroinflammation is a primary driver of alcohol-induced damage to the hippocampal CA1 and CA3 regions, but the involvement of additional mechanisms to fully elucidate the complexity of hippocampal injury under chronic alcohol exposure.

Studies have shown that alcohol exposure activates the immune response, partially activating astrocytes and microglia to promote the synthesis of inflammatory mediators [e.g., inducible nitric oxide synthase (iNOS), cyclooxygenase-2 (COX-2)] and induce the release of pro-inflammatory cytokines (IL-1β, TNF-α, IL-6) from glial cells. This cascade aggravates brain tissue inflammation, drives necrosis and structural abnormalities in CA1 and CA3 neurons, and ultimately results in hippocampal neuronal dysfunction (36, 43). Notably, the gut microbiota plays a pivotal role in this process: pro-inflammatory gut microbiota (e.g., excessive *Escherichia coli*) releases lipopolysaccharide (LPS), which translocates across a compromised intestinal barrier into the systemic circulation, reaches the hippocampus, and activates microglia via the TLR4/NF-κB pathway—amplifying pro-inflammatory cytokine production (IL-1β, TNF-α) in the hippocampus and synergizing with alcohol-induced local inflammation to exacerbate neuronal loss in CA1 and CA3 regions (43). Conversely, beneficial gut bacteria (e.g., Bacteroides, Lactobacillus) produce short-chain fatty acids (SCFAs) that cross the blood–brain barrier to inhibit microglial activation, reduce inflammatory factor release, and protect hippocampal neurons by enhancing synaptic plasticity and preserving mitochondrial function (44, 45). Moreover, gut microbiota dysbiosis may disrupt tryptophan metabolism, diminishing serotonin production—a neurotransmitter critical for hippocampal function—and further impairing learning and memory processes in the CA1 and CA3 regions.

### 2.3 Amygdala

The amygdala is a smaller amygdaloidal pillar structure consisting of 13 nuclei located deep in the anterior-inferior region of the temporal lobe, beneath the central cortex, and connected to the prefrontal cortex, hippocampus, septal nuclei and thalamus (4). The major components of the amygdala are the central-medial nucleus of the amygdala (CeA), the bed nucleus of the stria terminal (BNST), and the shell of the nucleus accumbens (ACB-sh). The amygdala plays an important role in identifying danger and self-protection. Among them, CeA is the key brain region for emotional and behavioral integration, and plays a core role in the physiological behavior of fear stimulation, stress stimulation, and drug stimulation (46). Chronic alcohol exposure affects anxiety-like behavior, mainly due to impaired neurotransmission in the CeA region of the amygdala (47).

Gabaergic neuronal transmission of CeA plays a key role in the impairment of neurotransmitter transmission in the CeA region of the amygdala caused by alcohol exposure (48). Research shows that the damage of the intestinal barrier by alcohol triggers a chain reaction, which in turn exacerbates the damage to the central nucleus of the amygdala (CeA). Specifically, after the intestinal barrier is damaged, lipopolysaccharide (LPS) produced by Gram-negative bacteria such as *Escherichia coli* and *Klebsiella* can enter the brain through the bloodstream and directly activate microglia and astrocytes in the CeA. Since the CeA highly expresses Toll-like receptor 4 (TLR4), LPS specifically binds to it and triggers the NF-κB signaling pathway, promoting the release of pro-inflammatory cytokines such as TNF-α and IL-1β, thereby inhibiting the basic transmission function of GABAergic neurons. At the same time, the imbalance of the intestinal microbiota caused by long-term alcohol exposure leads to the over-proliferation of *Enterococcus faecalis*. Its metabolites, lipoteichoic acid (LTA) and reactive oxygen species (ROS), further damage the integrity of the intestinal barrier and exacerbate the translocation of LPS. Among them, LTA can penetrate the blood–brain barrier and directly activate the astrocytes in the CeA, prompting them to release chemokines such as monocyte chemoattractant protein-1 (MCP-1), recruiting peripheral immune cells to aggregate towards the CeA, thus amplifying the neuroinflammatory response (49). Neuroimmune activation increases gabaergic inhibitory postsynaptic potential, increases the expression of postsynaptic gabaergic receptors, inhibits basal gabaergic transmission in CeA, and affects CeA neurotransmitter transmission (50). Therefore, alcohol-induced damage to the CeA region of the amygdala is mainly caused by the blockage of neurotransmitter transmission caused by neuroinflammation, resulting in emotional stimulation disorder.

### 2.4 Hypothalamus

The hypothalamus, located below the thalamus, is the centre of the autonomic nervous system and is divided into the crossover region (CR, chiasmatic region), the nodal region (IR, infundibulotuberal region) and the posterior region (MR, mammillary region). The hypothalamus is responsible for maintaining the basic homeostasis of the body’s neuroendocrine and visceral functions (51). Among them, the paraventricular nucleus (PVH), as an important part of the CR zone, plays an important role in promoting the secretion of corticotropin-releasing hormone (CRH) and in the regulation of neuroexcitability (52). Chronic alcohol abuse damages the hypothalamus, and further studies in male and female rats exposed to alcohol for 10–16 months report that hypothalamic neuronal loss occurs primarily at the PVH (53). Notably, beyond the well-characterized inflammatory pathways, emerging evidence suggests that autophagic dysfunction may also contribute to hypothalamic neuronal damage under pathological conditions. Studies have shown that davunetide promotes structural and functional recovery of injured spinal cords by enhancing autophagy, highlighting the potential of autophagy modulation in neuroprotective strategies. This raises the question of whether alcohol-induced PVH damage involves impaired autophagic flux, and whether targeting autophagy (e.g., via davunetide-like agents) could alleviate CRH neuron overactivation and restore visceral homeostasis—an avenue worthy of further investigation (54).

Alcohol damage to the PVH region of the hypothalamus is mainly caused by overexpression of CRH neurons (55). Studies have shown that chronic alcohol leads to the activation of the NF-κB pathway in PVH, which promotes the levels of inflammatory cytokines such as interleukin-1β (IL-1β), tumor necrosis factor-α (TNF-α), interleukin-6 and interleukin-18. It increases the excitability of peripheral sympathetic nerve and stimulates the secretion of corticotropin-releasing hormone (CRH), which leads to the overexpression of CRH neurons in the PVH area, and then leads to the abnormal regulation of visceral function (53). Importantly, emerging evidence reveals that alcohol-induced gut microbiota dysbiosis (characterized by increased *Enterococcus faecalis* and decreased *Akkermansia muciniphila*) compromises intestinal barrier integrity, facilitating lipopolysaccharide (LPS) translocation into circulation. The circulating LPS then activates the TLR4/NF-κB signaling pathway in PVH, exacerbating neuroinflammation and further promoting CRH neuron hyperactivation. This gut-brain axis mechanism provides a comprehensive explanation for alcohol’s detrimental effects on PVH function (14–16).

### 2.5 Cerebellum

The cerebellum is globular in shape and is located underneath the entire brain, behind the brain stem. The cerebellum is divided from the outer to the inner layers into: molecular, Purkinje and granular layers, which are mainly involved in the maintenance of balance, orientation, and motor (56). Among them, the Purkinje cells (PCs) in the Purkinje layer play a more important role as primary integrating neurons in the cerebellum in the regulation of motor (57). Cells in different layers of the cerebellum are differentially sensitive to chronic alcohol abuse, with the PC being the most vulnerable, as evidenced mainly by a general reduction in cell number and density. In animals receiving ethanol intervention, a balance beam assay was performed and found that ethanol-intervened rats had reduced balance and showed PC neuronal death through reductions in neuronal number and density (58).

Alcohol exposure-induced apoptosis of PC neurons important cause of cerebellar damage, study suggests. In UChB rats, ethanol intake induced the release of cerebellar microglia, which led to a significant increase in the expression of caspase-3 and XIAP genes, which were responsible for cell apoptosis (59). Alcohol exposure alters GABA receptor-dependent neurotransmitters. GABA release was found to increase in the PC layer after alcohol treatment as well as in interneurons and granule cells in the PC molecular layer (60). It causes the transmission disorder of the related synaptic parts of PC neurons and the sensitivity of neurons to alcohol toxicity, which increases the number of caspase-3 positive cells and apoptosis rate in the PC region, indicating the apoptosis of PC neurons and leads to abnormal cerebellar motor function (61). Notably, the gut microbiota exerts a pivotal regulatory role in modulating the damage or protection of cerebellar Purkinje cells (PCs) via the gut-brain axis. Bacteroides and their metabolic byproducts (e.g., polysaccharide A derived from *Bacteroides fragilis*) confer neuroprotective effects on PCs through multifaceted mechanisms: they inhibit microglial activation via the TLR2 signaling pathway, thereby attenuating neuroinflammatory responses; regulate microglial phenotypic polarization to suppress the pro-inflammatory M1 subtype, mitigating neurodegenerative damage; and enhance synaptic plasticity through short-chain fatty acids (SCFAs) (e.g., butyrate) that act on GPR41/43 receptors, collectively reducing apoptotic stress on PCs (44). In contrast, excessive proliferation of *Escherichia coli* exacerbates PC injury by activating caspase-3-dependent apoptotic cascades: its toxins (e.g., Shiga toxin) synergize with caspase-3 to cleave gasdermin D (GSDMD), promoting pyroptotic cell death, while *E. coli*-derived LPS triggers the Caspase-11/GSDMD pathway to amplify neuroinflammation, further compromising PC viability (62). Additionally, gut microbiota dysbiosis impairs cerebellar energy metabolism and PC function by reducing vitamin B1 (thiamine) synthesis (45). As a critical coenzyme for the pyruvate dehydrogenase complex, thiamine is indispensable for maintaining mitochondrial function; its deficiency disrupts PC electrical activity and survival, exacerbating functional deficits in these neurons.

**Figure 1:**
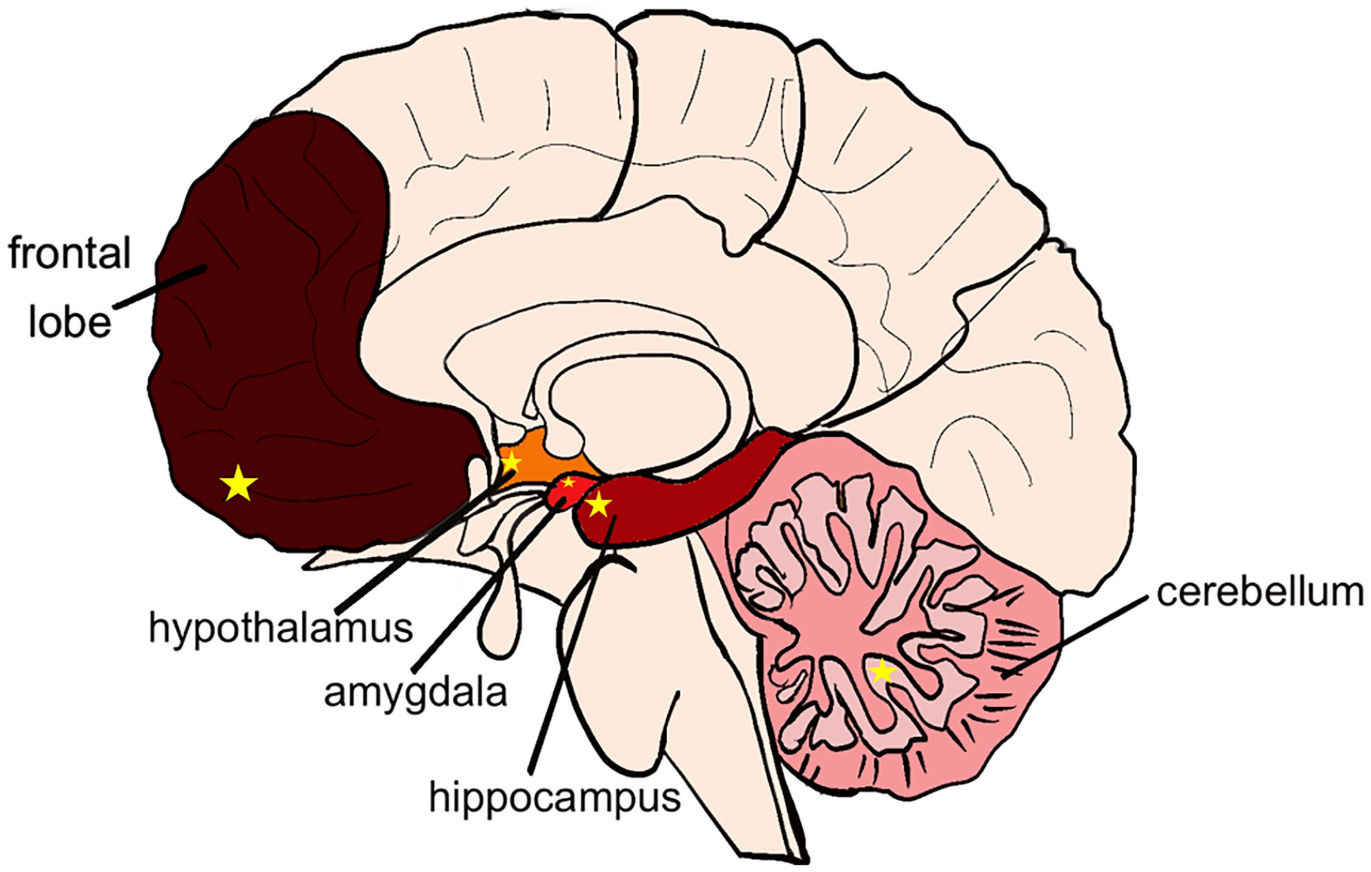
Distribution of alcoholic brain injury. Color from dark to light indicates the degree of alcohol damage: frontal lobe > hippocampus > amygdala > hypothalamus > cerebellum. Yellow star in the frontal lobe: PFC area. Yellow star in the hippocampus: CA1 and CA3 area. Yellow star in the amygdala: CeA area. Yellow star in the hypothalamus: PVH area. Yellow star in the cerebellum: PC area.

Alcohol can cause brain injury and dysfunction in decision-making, cognition, and motor functions, and different parts of the brain are differently sensitive to alcohol, and the corresponding subregions show different alcohol sensitivity, there is a close association between the effects of alcohol on different regions of the brain and the gut microbiota. Alcohol-induced gut microbiota dysbiosis can through multiple pathways including endotoxin translocation, short-chain fatty acid metabolic disorders, and vagus nerve signal transduction, mediate functional damage in specific brain regions such as the prefrontal cortex, hippocampus, and paraventricular nucleus of the hypothalamus. This suggests that the composition of the microbiota may be one of the key regulatory factors determining the differences in alcohol sensitivity among different regions of the brain. Frontal lobe changes are more pronounced in patients with chronic alcohol disorders, as measured by neuropathologic magnetic resonance imaging (63). Meta-analysis revealed that gray matter in prefrontal cortex, hippocampus, hypothalamus, and cerebellum in patients with chronic alcohol disorders showed reduced gray matter, and the reduced gray matter density in key nodes of the synaptic network was associated with patients’ reduced attention and working memory capacity. Further studies have found that the frontal lobes (especially the prefrontal cortex) are more susceptible to damage in patients with chronic alcohol disorders (64). In addition, the brain magnetic resonance imaging to observe the gray matter (GM) and white matter (WM) areas of different brain regions in alcoholics, and found that larger and more significant clusters were located in the prefrontal region. Based on morphometry, the volume of prefrontal lobe, hippocampus, amygdala, thalamus and cerebellum cluster was found to be less and less affected by alcohol (65). At present, there are relatively few comprehensive studies on the extent of alcohol damage to other parts of the brain and the relationship between alcohol-induced dysfunction and various parts of the brain. Emerging evidence shows that alcohol, through the complex gut-brain axis, has different effects on different regions of the brain, and these effects are closely related to gut microbiota dysbiosis. The imbalance of gut microbiota caused by alcohol, manifested as an abnormal ratio of Firmicutes/Bacteroidetes, can disrupt the normal functions of specific brain regions such as the prefrontal cortex, hippocampus, and paraventricular nucleus of the hypothalamus. Based on the above, we can conclude that alcohol has the most severe damage to the frontal lobe (PFC), which shows the most severe symptoms of decision-making and attention disorders. Followed by hippocampus (CA1 and CA3 areas), amygdala (CeA area), hypothalamus (PVH area) and cerebellum (PC area). In turn, the disorders were cognitive memory, emotion, endocrine and motor regulation (Figure 1). In the course of discussion, it is considered that the site of injury may be affected by factors such as different acute or chronic patterns of alcohol intake, varying ages at which alcohol intake begins, differences in the degree of alcohol metabolism in the human body, dietary structure, individual variations in alcohol sensitivity, and the structural plasticity of nerve cells (66). Although previous studies have found an association between the microbiota and damage in different parts of the brain, the extent of the impact of microbiota diversity and types on the brain remains unclear. Therefore, future research needs to systematically explore alcohol-mediated brain injury regions based on variable factors such as the degree of injury in different brain regions, alcohol intake, gender, age, and diet, so as to better guide research on the targeted regions of alcohol-induced brain dysfunction.

## 3 Mechanism of action of alcohol-induced brain injury to learning and memory dysfunction based on the gut-brain axis theory

Previously, we discussed and concluded that the direct causation of brain injury is a combination of inflammation, oxidative stress, and neurotransmitter cross-linking. But through research reports we have found that these mechanisms are not the starting point of brain injury caused by alcohol. Transplantation of gut microbiota from alcohol-fed mice into normal healthy controls significantly shaped gut bacterial composition and elicited behavioral signs of alcohol withdrawal induced anxiety, demonstrating that changes in the gut microbiota environment are capable of altering brain health (7). In general, it is believed that alcohol can spread alcohol and some metabolites in the intestine outwards by destroying the balance of gut microbiota and the integrity of intestinal shielding, which promotes the subsequent cascade reactions with the nervous system, immune system and endocrine system, and then damage the central nervous system and cause brain dysfunction (67).

### 3.1 Gut microbiota and the nervous system

The nervous system is divided into the central nervous system, which consists of the brain and spinal cord, and the peripheral nervous system, which consists of cerebral nerves connected to the brain and spinal nerves connected to the spinal cord (68). The enteric nerve belongs to the peripheral nervous system, and the vagus nerve as a parasympathetic nerve also belongs to the peripheral nervous system. The vagus nerve is the main bidirectional connection channel between the enteric nervous system and the central nervous system, including vagal afferent fibers and efferent fibers. It travels from the brain through the trunk to the intestine and the internal organs of the body, and the signal is transmitted throughout the nervous system by neurotransmitters (69). Gut microbiota can directly produce 5-HT, LPS and other products, which stimulate the central nervous system through the afferent fibers of the vagus nerve and eventually act on the brain. After the brain signals to immune cells through efferent fibers, it promotes the inactivation of pro-inflammatory M1 macrophages and inhibits the release of inflammatory factors such as IL-1β and IL-6, thereby reducing intestinal inflammation (70). Studies based on the gut-brain axis theory have found that alcohol can cause direct or indirect effects on neurotransmitters and their transmission through gut microbiota (71). Chronic alcohol action decreases the abundance of beneficial bacteria such as *Lactobacillus* spp. and *Bifidobacterium bifidum*, and directly decreases neurotransmitters such as glutamic acid (Glu), gamma-aminobutyric acid (GABA), and 5-hydroxytryptamine (5-HT) (14–16, 72). In addition to this, alcohol altering the gut microbiota may also affect neurotransmitters through inhibition of the vagus nerve, and the vagus nerve plays an important indirect effect in this process. Absence of neurochemical and behavioral effects found in mice with removal of the vagus nerve (33). Notably, modulating METTL14 and BDNF via traditional medicine has been shown to mitigate neural damage, highlighting potential therapeutic targets for alcohol-related brain injury (73). When the vagus nerve is removed, substances such as 5-HT and cholecystokinin produced by alcohol-affected enteroendocrine cells will not be able to pass messages through the vagus nerve to the central nervous system to act on the brain (74). It also fails to activate the downstream inhibitory pathways in the brainstem, blocking the release of inhibitory neurotransmitters such as GABA within the dorsal horn of the spinal cord, and cannot serve to regulate intestinal inflammation (75).

Based on the deepening of the theory of the gut-brain axis and the extensive research on the modulation of neurological functions by intestinal bacteria, we have found that gut microbiota and their metabolites can play an important role in many neurological disorders of the brain, such as autism and memory loss (76). In particular, relevant gut microbiota capable of producing neurotransmitters and their metabolites are of great significance for the modulation of neural pathways in entero-brain injury (77). However, there have been few studies on the isolation and identification of this neurotransmitter-producing gut microbiota. At the same time, in the gut-brain axis after alcohol injury, the specific gut microbiota that regulates neurons, the signals transmitted and the specific regulatory mechanism are not clear. In the future, more detailed studies will be conducted on the mechanism of alcohol-induced brain nerve injury, and indirect intervention will also be carried out through the screening of gut microbiota producing specific metabolites.

### 3.2 Gut microbiota and the immune system

In alcohol-induced brain injury, the gut microbiota serves as a critical mediator in triggering neuroinflammation. The immune system plays a pivotal role in this cascade by sensing microbial alterations in the gut and transducing these signals into inflammatory or immunoregulatory responses that affect the central nervous system. Immune cells exist in the gut in the form of T cells, while in the central system, microglia are both nerve cells and immune cells (78). Intestinal T cells and brain microglia have a necessary connection. Exogenous stimulation changes gut microbiota and metabolites, and its activity information is rapidly transmitted to the nervous system through the intestinal immune system (79). A comprehensive analysis of the process by which alcohol targets gut microbiota and causes alcohol brain damage by activating the gut-brain immune system is as follows: according to the previously mentioned cases of brain injury caused by fecal transplantation, alcohol addiction or withdrawal alters the composition and abundance of gut microbiota, resulting in an increase in harmful bacteria such as *Firmicutes*, *Proteobacteria*, and *Escherichia coli* (80). It also causes a decrease in beneficial bacteria such as *Mycobacterium anisopliae*, *Lactobacillus*, *Prevotella*, etc. (81) where harmful bacteria such as *Aspergillus* enhance intestinal permeability through direct disruption of tight junctions, production of toxins or proteases, and activation of inflammatory cascade reactions (82). At the same time, with the increase of the harmful metabolite LPS and the decrease of the beneficial metabolite short-chain fatty acids (SCFAs), metabolites entering the systemic circulation stimulate the differentiation of T lymphocytes in intestinal immune cells, leading to the proliferation of intestinal immune effector cells and the stimulation of microglia in central immune cells through the blood (83). In this process of the gut-brain immune system T cells and microglia work together by activating signaling pathways such as PI3K/AKT/mTOR, MAPK, NF-κB, etc. Causes the release of inflammatory factors such as interleukin-1β (IL-1β), interleukin-6 (IL-6) and tumor necrosis factor-α (TNF-α) (40, 41). Induces disorders of the central nervous system, ultimately affecting brain functions such as learning and cognition (9, 84), the above are central immunological processes.

The immune system is divided into central and peripheral immunity on the basis of its cascade with the central and peripheral nervous systems, which are interconnected through the blood circulation (including the somatic circulation) and the lymphatic circulation (85). Current research related to brain injury caused by alcohol via the neuroimmune pathway focuses on the link between enterocytes and the central nervous system and is achieved through the somatic circulation (83). Studies of alcoholic brain injury involving central immunity are more complete, while fewer studies have been conducted on peripheral nervous system-related immune responses. Studies have shown that when the blood of the human body flows through the capillaries, some water and small molecular proteins can enter the tissue fluid through the capillaries. A small amount of tissue fluid enters the lymphatic capillaries and merges with lymph fluid to form the common lymphatic vessels through the lymphatic circulation. Finally, it is injected into the superior and inferior vena cava of the body, so that it is returned to the blood circulation. So the lymphatic circulation and the blood circulation intersect (86). This may be related to the involvement of immune cells in the lymphatic circulation. Based on previous studies, a small proportion of the osmotic fluid in the blood can circulate through the lymph to the blood. We speculate that alcohol-induced peripheral immunity is due to the infiltration of some intestinal metabolites such as LPS into the lymph fluid, which may stimulate the proliferation and division of B lymphocytes and the secretion of antibodies. Antibodies are formed by circulating with body fluids and acting on the peripheral nervous system (87). Based on the above, it is reasonable to speculate that there may be a cascade relationship between alcohol-induced central immunity and peripheral immunity. In the future, based on the theory of central immunity, peripheral immunity and central-peripheral immunity can be studied for the regulatory mechanism of brain injury.

### 3.3 Gut microbiota and the endocrine system

The endocrine system is composed of endocrine glands and endocrine cells. Intestinal epithelial cells (IECs), which include hormone-secreting enteroendocrine cells (EECs), are considered important regulators of metabolism and immunomodulation. When chronic alcohol acts on gut microbiota, it will reduce the production of beneficial metabolites of gut microbiota (such as SCFAs, tryptophan, etc.), and reduce the secretion of gastrointestinal hormones and peptides by EECs (88). These secretions are absorbed and enter the circulation, and the decrease in secretions inhibits activation of peripheral immune cells. Among them, the production of interferon-γ (IFNγ) by activated CD4 cells (Th1), CD8 cells and NK cells decreased. Depletion of interferon-γ inhibits the expression of anti-inflammatory TRAIL in astrocytes, which in turn inhibits TRAIL-DR5 signaling. It can promote neuroinflammation, change the integrity of the blood–brain barrier (BBB), and indirectly affect the activity of central neurons (11, 89). When the neuroendocrine cells in the medial small cells of the paraventricular nucleus (PVN) are stimulated by neuroinflammatory factors (TNF-α, IL-1, IL-6), the hypothalamic-pituitary-adrenal axis (HPA axis) is activated, which increases the susceptibility to exogenous stress. The hormones released during this process send signals down through the body fluids, for example, the PVN releases large amounts of corticotropin-releasing hormone (CRH), which prompts the release of adrenocorticotropic hormone (ACTH) from the anterior pituitary gland. The adrenal cortex can synthesize glucocorticoids under the action of ACTH (35). The increase of glucocorticoids can promote gastric acid secretion and reduce gastric mucus secretion, resulting in vasospasm of the gastrointestinal tract, necrosis of the gastric mucosa, and affecting intestinal function (90).

**Figure 2:**
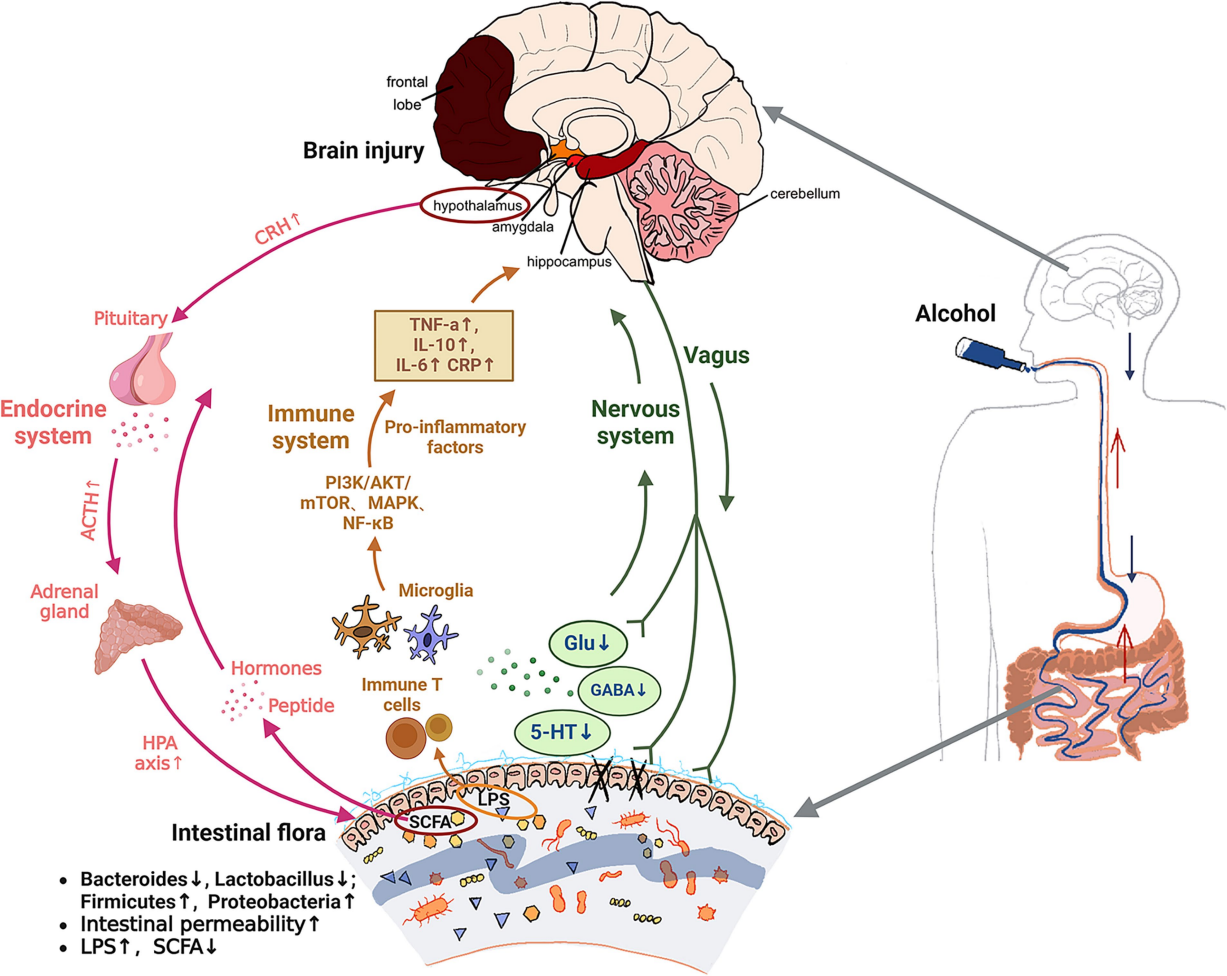
Mechanism of alcohol induced brain injury based on gut brain axis theory. Green arrows: neural pathway. Brown arrows: immune pathway. Pink arrows: endocrine pathway. ↑, increase; ↓, decrease; Glu, glutamic acid; GABA, γ-aminobutyric acid; 5-HT, 5-hydroxytryptamine; TNF-α, tumor necrosis factor-α; IL-10, interleukin-10; IL-6, interleukin-6; CRP, C-reactive protein; LPS, lipopolysaccharides; SCFA, short chain fatty acid; CRH, corticotropin releasing hormone; ACTH, adrenocorticotropic hormone; HPA axis, the hypothalamic-pituitary-adrenal axis.

Based on the above discussion of alcohol-induced brain injury, it is found that the nervous (green arrows in Figure 2), immune (brown arrows in Figure 2) and endocrine systems (pink arrows in Figure 2) that constitute the microbiota gut-brain axis are highly complex but interrelated. Alcohol is ingested orally into the gastrointestinal tract through the oesophageal and acts on gut microbiota in the intestinal tract. Increasing harmful bacteria such as *Firmicutes* and *Escherichia coli* that can produce harmful metabolites LPS, and reducing beneficial bacteria such as *Lactobacillus* and *Bifidobacterium* that can produce beneficial metabolites such as SCFA and neurotransmitters (Glu, GABA and 5-HT, etc.). First of all, the reduction of neurotransmitter content will induce the nervous system to respond. The damaged intestine will send signals to the central system through the vagal afferent fibers, and the central nerve will also affect the intestinal permeability through the vagal efferent fibers (neural pathways). In addition, as the harmful metabolite LPS increases and enters the systemic circulation, it stimulates intestinal immune T cells and central microglia, activates PI3K/AKT/mTOR, MAPK, NF-κB and other signaling pathways, produces inflammatory factors such as TNF-α and IL-10, induces central nervous system disorders and causes brain injury (immune pathway). With the reduction of the beneficial metabolite SCFA, the gastrointestinal hormones and peptides secreted by enteroendocrine cells are reduced into the blood circulation. By inhibiting interferon-γ (IFNγ) signaling, it alters the integrity of the blood–brain barrier (BBB) and indirectly affects the activity of central neurons. The hypothalamic paraventricular nucleus (PVN) neuroendocrine cells are stimulated, the HPA axis is activated, and a large number of CRH and ACTH are emitted through the body fluids. The glucocorticoids produced ultimately affect gut health (endocrine pathway). There may be a cross-link between these three pathways. The endocrine system reflects the connection between the endocrine system and the immune system through the blood–brain barrier caused by peripheral immune inflammation, and both the immune system and the endocrine system ultimately feedback to the brain through the central nervous system. Given the complex network of gut-brain interactions, it is anticipated that improving alcohol-mediated learning and memory impairment will require addressing gut microbiota. In addition to enhancing the proportion of the abundance of beneficial strains of gut microbiota, improving the intestinal environment, and reducing intestinal permeability, from the perspective of the three regulatory pathways, it is also feasible to focus on screening specific gut microbiota and their metabolites that regulate neurotransmitters. And the mechanism of LPS flowing through lymphatic circulation acting on peripheral nervous system to generate peripheral immunity (91), to act on the gut-central-brain link to improve learning and memory dysfunction. At the same time, we found that some natural active substances can better regulate gut microbiota, which can be used as a way to solve alcohol-induced brain injury in the future.

## 4 Natural active substance improve alcohol-cognitive impairment through the gut-brain axis

With a lot of research on the intestinal brain axis theory in brain injury, more and more scholars focus on the natural active substances that can change the intestinal flora to regulate the brain and behavior, and then solve the problem of diseases caused by cognitive impairment. Polysaccharides, polyphenols, saponins, flavonoids and other natural active substances according to their different biological activity, in the form of human food, through the nervous system, immune system and endocrine system regulate intestinal flora composition and abundance, regulate metabolites, protect the shielding integrity, eventually improve the brain injury (Table 1). In addition, comparative analyses of *in vivo* experiments and computational simulations in traumatic brain injury (TBI) models have revealed that the choice of principal strain rate calculation method significantly influences the localization of brain injury regions. These findings suggest that distinct brain regions may exhibit differential sensitivity to external interventions (110). Therefore, the research and application of these natural substances will undoubtedly have a profound impact on many fields.

**Table 1:** Natural actives improve changes in the gut microbiota of brain injury.

Categories	Source	Structure	Experimental model	Changes in intestinal flora	Mechanism of improvement	Literature sources
Polysaccharides	Pu-erh tea polysaccharide	Structural formula: 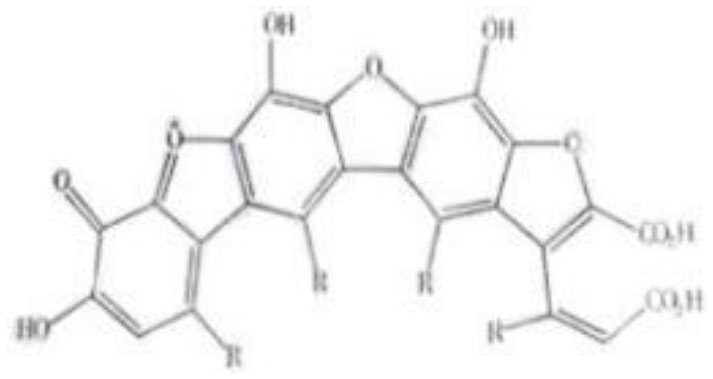 Molecular weight: 251.2 kDa Main chain: Beta-pyran glycosidic linkage	Mice with colitis	Beneficial: Bacteroidetes↑ Harmful: Firmicutes↓, Proteobacteria↓, *Helicobacter pylori* and faecalibacterium↓	Lactobacillus → restoring the contents of neurotransmitters Glu and 5-HT → improving the balance of neurotransmitters → restoring the homeostasis of the nervous system → reducing the patients’ emotional depression and anxiety behaviors	Xu et al. (105)
	Crassostrea crassostrea polysaccharide	Molecular weight: 623.24 kDa Main chain: β-pyranoglycosidic linkages	Alcohol-induced liver injury in mice	Beneficial: Reuteri Picea↑, Bacteroides↑		Jiang et al. (106)
	Fu brick tea polysaccharide	Molecular weight: 335.68 kDa Main chain: Alpha and beta type pyranoglycosidic linkages	Patients with inflammatory bowel disease	Beneficial: Bacteroides↑, Escherichia↑, Parabacteroides and Parasarteria↑	Bacteroides → regulating GLP1 and peptide YY secreted by intestinal endocrine cells → acting on the central nervous system → improving the blood–brain barrier Bifidobacterium can upregulate BDNF, promote the release of neurotransmitters, enhance the plasticity of enteric nervous system, improve the upward neural pathway, and exert positive effects on emotional anxiety and depression	Wang et al. (107)
Saponins	Ginsenosides	Structural formula: 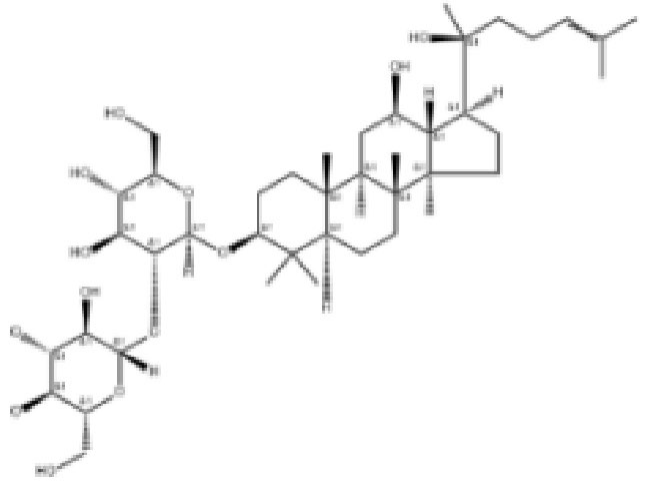 Molecular weight: 785.03 Da Main chain: Four benzene rings	Diabetic mice	Beneficial: Bifidobacterium↑, Proteus↑, Lactobacillus↑ Harmful: TM7↓		Yu et al. (22)
Polyphenols	EGCG	Structural formula: 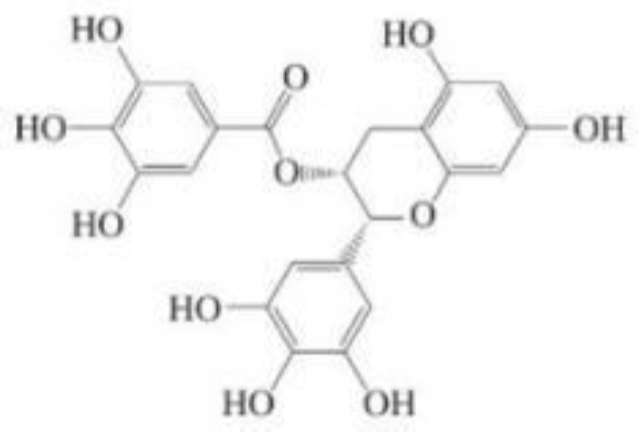 Molecular weight: 458.38 Da Main chain: Two aromatic rings and one pyran ring	Intestinal injury in mice with cyclophosphamide (CTX)	Beneficial: Rodentiaceae↑ Harmful: Pilospirillaceae and Desulfurovibriaceae↓	Prevotella → reduces the level of inflammatory factor IL-1β → improves neuroinflammation → improves central immune pathways → improves brain damage	Wei et al. (108)
	Polyphenols of oolong tea	Structural formula: 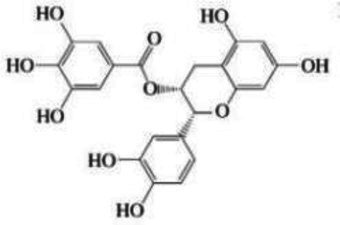 Molecular weight: 281.36 Da Main chain: Two aromatic rings and one pyran ring	Healthy volunteers	Beneficial: Lactobacillus↑, Bifidobacterium↑, Prevotella↑ Harmful: Clostridium histolytica↓		Sun et al. (109)

↑, the number of bacteria increased; ↓, the number of bacteria decreased; Glu, glutamic acid; 5-HT, 5-hydroxytryptamine; GLP-1, glucagon-like peptide 1; BDNF, brain-derived neurotrophic factor; EGCG, epigallocatechin gallate sulfate; IL-1β, interleukin-1β.

Polysaccharides, as natural high molecular weight polymeric carbohydrates, are usually not easily digested and absorbed by the small intestine, but they can promote the selective growth of a variety of beneficial bacteria in the large intestine to form a dominant flora and inhibit pathogenic bacteria (92). Polysaccharides can also be used as a carbon source for the gut microbiota in the intestinal fermentation and decomposition, maintaining physiological activity and regulating the composition of the gut microbiota to improve the health of the body (93). Comprehensive studies suggest that polysaccharides may ameliorate brain injury through the gut-brain axis from both the nervous system and the endocrine system, and that the activity of a polysaccharide depends on its structural properties. The molecular weight of the polysaccharide was found to be an important factor in activity, with fractions with molecular weights around 100,000 Da being more active, while fractions with molecular weights of 5,000–10,000 Da from the same source were inactive (94). However, it has also been found that mushroom polysaccharides with high molecular weights (1,160,000–1760,000 Da) show less activity than those with 301,000 Da. Therefore, it is believed that polysaccharides with moderate molecular weights show the best activity, while the optimal molecular weight ranges of different polysaccharide sources are also different. The number of branches also affected the polysaccharide activity, and the presence of branches at C-4 of the glucopyranose group linked by water-soluble β-1, 3-glucan was found to be significantly more active than that without branches (95). Meanwhile, lentinus mushroom polysaccharide L2 with a molecular weight of 26 KDa and a 1 → 3 linkage side chain could up-regulate *Bacteroides* in beneficial bacteria in the gut. The production of SCFAs regulates GLP1 and peptide YY secreted by intestinal endocrine cells and acts on the central nervous system to improve the integrity of the blood–brain shield (96). In addition, polysaccharides containing specific β-(1 → 3)-glucan side chains have been shown to modulate beneficial gut bacteria such as *Bacteroides*, thereby influencing the production of gut-derived hormones and neurotransmitters. This may, to some extent, affect brain regions closely associated with cognitive function, including the prefrontal cortex and hippocampus. Although this hypothesis has begun to emerge in animal studies, direct evidence linking polysaccharide intervention to specific brain regions remains limited, and the underlying mechanisms are still poorly understood. Future research should focus on elucidating the relationship between the structural specificity of polysaccharides and the functional modulation of targeted brain regions. This would not only deepen our understanding of the fine-tuned regulation within the gut–brain axis in cognitive disorders, but also provide a theoretical foundation for the precision application and clinical translation of natural polysaccharide-based interventions.

Natural active substances with relatively small molecular weights (about 1,000 Da), such as saponins, which are composed of aglycones and sugar groups. The molecular weight of saponins and the number of glycosyl branches affect their activity. It was found that platycodin PE, with a molecular weight of 700–1,000 Da, exhibited stronger saponin activity when it had only one more terminal furan group at C-28 position than platycodin DPE (97). The increased activity was associated with increased branching of glycosyl moieties as well as with the type of glycosyl moieties. Many saponins may not be directly absorbed and utilized, and gut microbiota can modify saponins. Gut microbiota dehydroxylated saponins to form double bonds and rare saponins, thereby exerting anti-inflammatory activity through changes in the abundance of gut microbiota. Ginsenoside is converted to ginsenoside Rd, Rh2, CK, PPD and other products under the action of gut microbiota. Among them, Rh2 with diol structure, C-3 and C-20 attached glycosyl branches and molecular weight of 785.03 Da can significantly increase IL-2 activity. It can also act as an immunomodulatory factor to promote the synthesis of albumin and γ-globulin, improve the function of T cells and macrophages, inhibit chromosome mutations in lymphocytes, and stabilize the immune system (98). Therefore, gut microbiota as a mediator play an important role in the improvement of natural saponins.

Polyphenols with smaller molecular weight (less than 600 Da) can not only directly enter the blood stream, but also be improved through the BBB by secretase in gut microbiota, which can be converted into bioactive small molecular metabolites that are more easily absorbed by the body (99). Resveratrol, which is made of benzene ring and phenylpropane ring in nature, has two hydroxyl groups on the benzene ring and one hydroxyl group on the phenylpropane ring (100). These hydroxyl groups are able to bind to free radicals and form stable radical complexes. These naturally stable complexes cannot be directly used by the human body and can be metabolized and absorbed by gut microbiota. Resveratrol was metabolized by gut microbiota to dihydroresveratrol, which reversed the ratio of *Firmicutes* to *Bacteroidetes* and increased the abundance of *Prevotella* in the gut (101). The abundance of *Prevotella* was negatively correlated with interleukin-1β (IL-1β) level (67). Therefore, polyphenols can be used to increase the abundance of *Prevotella* through gut microbiota metabolism, reduce the level of inflammatory factor IL-1β, and improve the effect of neuroinflammation on the central immune pathway, which plays a neuroprotective role and improves brain injury. Studies have also found that different polyphenol arrangement has different activity, and trans resveratrol with aromatic ring, phenolic hydroxyl group and double bond structure is more stable than cis resveratrol (24). It is concluded that polyphenols with trans spatial structure and hydroxyl branch can enrich *Prevotella* to improve neuroinflammation and regulate the immune system of gut-brain axis.

Recent preliminary clinical studies have provided evidence that certain natural bioactive compounds can improve cognitive function by modulating the gut microbiota. For instance, ginsenoside Rh2 has shown promise in individuals at high risk of Alzheimer’s disease, potentially ameliorating central inflammation and cognitive decline through immune modulation along the gut–brain axis (98). Additionally, resveratrol has been demonstrated to enhance plasma levels of SCFAs and improve working memory performance (101). These findings provide preliminary clinical support for the potential of natural compounds to modulate gut microbiota and improve cognitive impairment. However, large-scale, multicenter clinical trials are warranted to validate these effects and establish a mechanistic link between gut microbial changes, central neuroinflammation, and cognitive outcomes.

Interestingly, neuro-natural drugs or compounds follow the concept of stimulants, which is a biphasic dose–response process (18, 19). Through this process, low/moderate doses of stimulant drugs and/or natural compounds can induce cellular adaptive responses, upregulate cellular elastin, activate antioxidant pathways (Nrf2, SOD, GSH, HO-1) to induce health benefits, thereby enhancing brain health during neurological diseases (18). Conversely, high doses of drugs or compounds may exert neurotoxicity on cells and tissues, leading to increased oxidative stress markers and subsequent suppression of antioxidant proteins, thereby contributing to the onset and progression of neurological disorders (19). Similarly, the ethanol-induced dose–response relationship, analogous to that of stimulants, can be described as having a biphasic effect on brain cells indicate that ethanol exhibits excitatory effects on astrocytes at low doses, with a biphasic dose–response curve observed across the entire ethanol concentration range (102). However, high-concentration ethanol directly disrupts the lipid bilayer structure of cell membranes. It inhibits NMDA receptor function while enhancing GABA receptor function, causing severe disruption of neural communication and triggering significant oxidative stress and inflammation in the brain. Interestingly, studies have shown that oral low-dose (10 or 1 mg/kg) curcumin lipid-core nanocapsules exhibit stronger neuroprotective effects compared to high-dose free curcumin (50 mg/kg) by reducing Aβ1–42-induced inflammatory cytokine levels (such as TNF-α, IL-6, IL-1β, and IFN-γ) in the serum and prefrontal cortex and hippocampus of aged mice (18). Currently, the field focusing on stimulant/adaptive responses activated by drugs or natural compounds—which enhance the endogenous redox defense system—is emerging as a promising preventive and therapeutic strategy for neurological disorders, including major depressive disorder.

By summarizing the relationship between the structure, efficacy of natural active substances and gut microbiota, it can be concluded that it is feasible to use natural active substances to change the gut microbiota to affect three pathways in the improvement of alcohol-induced cognitive impairment. At the same time, according to the complexity of the structure of natural substances, moderate molecular weight, trans space, β-(1 → 3) glucan side chain branch structure will increase the beneficial bacteria of gut microbiota. The structure of functional groups such as furanose group and hydroxyl group will be metabolized by bacteria to produce a more active configuration, of course these are also affected by different factors such as the source of the natural substance, metabolites and so on (103). Therefore, the optimal active fraction can be screened by structural identification in the future. It can also be structurally modified by physical and chemical means to improve brain injury by acting on gut microbiota with higher biological activity. In addition, the abundance of *Prevotella* is negatively correlated with interleukin-1β (IL-1β) levels and may influence brain injury affected by central immune pathways (67). It was found that different bacteria had different effects on alcoholic brain injury. In the future, we can develop targeted health products based on different microbial improvement mechanisms, or combine several bacterial groups to study their mixed health effects. Natural substances have the advantages of extensive biological activity, good effect and low resistance. This is different from the effect of direct consumption of “probiotic” freeze-dried powder. One study conducted endoscopic sampling of subjects taking probiotic supplements found no significant changes in the composition of gastrointestinal mucosa and lumen flora, which proved that direct consumption of probiotics could not be determined and played a role in the gut (104). Natural substances as prebiotics can play a protective role in three ways by regulating the metabolism of gut microbiota. Therefore, it has a broad application prospect in the treatment of chronic alcohol-induced memory and cognitive impairment.

## 5 Summary and outlook

Brain injury caused by chronic alcohol has become an important cause of brain diseases and behavioral disorders. Due to the intricate physiological structure of the human brain, different parts of the brain are affected by alcohol differently, and of course there are individual differences. A large proportion of these studies have found alcohol damage to the frontal lobe and hippocampus, and behavioral and histopathological experiments have evaluated the reduction of decision-making, cognition, and memory abilities in these patients. With the deepening of the theory of gut-brain axis, it is more believed that alcohol-induced brain injury is initiated by gut microbiota and cross-linked through three possible mechanisms of gut-brain axis: nerve, immune and endocrine: the inhibition of nervous system transmitters is regulated up and down by the vagus nerve, which stimulates the immune cells of the immune system to produce neuroinflammation and act on the central nervous system, and the intestinal endocrine disorders caused by the metabolism of hormones by the endocrine system bacteria cause peripheral immune inflammation to destroy the blood–brain barrier (BBB) and activate the HPA axis, thus affecting the host’s brain and behavior. Based on the previous discussion, there is a cross-link between the nervous, immune and endocrine systems in the regulation of the gut-brain axis. The immune system is connected with the central nervous system through the systemic circulation, which leads us to think about the lymphatic circulation between the peripheral nervous system and the immune system. At the same time, the link between the upward regulation mechanism of the endocrine system and the involvement of metabolites in the lymphatic circulation is also considered. Future research on the cross-link between peripheral immunity, endocrine and lymphatic circulation is expected to achieve a breakthrough in the regulation of brain injury based on the gut-brain axis. Starting from the improvement of gut microbiota, safer natural active substances with low drug resistance are used to solve the problem of learning and memory disorders caused by alcoholic brain damage. The moderate molecular weight, functional group (branching structure such as sugar group and hydroxyl group) and spatial structure (trans structure, branching structure of β-(1 → 3) glucan side chain) of polysaccharides, saponins and polyphenols can be used to increase the abundance of beneficial bacteria, so as to improve learning and cognitive impairment by regulating the nervous, immune and endocrine systems.

Although existing reviews have summarized the potential of natural bioactive substances to improve alcohol-induced brain injury through the gut-brain axis and their molecular mechanisms, significant limitations remain in this field: first, the depth of mechanism studies is insufficient. Macromolecules such as polysaccharides and saponins have relatively low absorption and conversion efficiencies, and their truly active forms and targets of action remain unclear. Human bioavailability, metabolic pathways, and interactions with the microbiota remain unclear. Second, clinical translational evidence is scarce. Most of the data come from animal models; current research is mainly focused on the preclinical stage, and there is a lack of large-scale randomized controlled trials to verify its efficacy and safety. Third, alcohol-induced damage mechanisms and severity vary across brain regions (e.g., prefrontal cortex, hippocampus, amygdala), yet the regionally selective protective effects of natural bioactive compounds and their underlying pathways remain unelucidated. Future research should prioritize: (1) Deepening mechanistic understanding using cutting-edge technologies like organoids and single-cell sequencing; (2) Conducting meticulously designed clinical trials to establish dose–response relationships; (3) Integrating neuroimaging techniques to assess region-specific protective effects; (4) Developing multi-omics integrated analysis methods to systematically reveal the multi-system regulatory networks of the gut-brain axis, providing theoretical foundations for precision interventions.
